# A Systematic Review of Major Cardiovascular Risk Factors: A Growing Global Health Concern

**DOI:** 10.7759/cureus.30119

**Published:** 2022-10-10

**Authors:** Dipannita Adhikary, Shanto Barman, Redoy Ranjan, Hana Stone

**Affiliations:** 1 Faculty of Life Sciences and Medicine, King’s College London, London, GBR; 2 Cardiovascular Science, Impulse Hospital, Dhaka, BGD; 3 College of Medicine, Mugda Medical College and Hospital, Dhaka, BGD; 4 Cardiac Surgery, Bangabandhu Sheikh Mujib Medical University, Dhaka, BGD; 5 Institute of Cardiovascular Research, Royal Holloway University of London, London, GBR; 6 Surgical Science Programme, The University of Edinburgh, Edinburgh, GBR

**Keywords:** awareness of cardiovascular disease, traditional cardiovascular risk factors, global burden, risk factors of cardiovascular diseases, cardiac risk factors and prevention

## Abstract

Cardiovascular disease has become a growing global and public health concern among non-communicable diseases (NCDs). The purpose of the study was to focus on the increasing prevalence of the risk factors of cardiovascular diseases (CVD), irrespective of age and gender, and its effect on public health worldwide. A literature search was done in the electronic database: Medline, PubMed, Web of Science, Google Scholar, and the World Health Organization (WHO) website, based on recent research and the prevalence of the risk factors of cardiovascular diseases. Moreover, a manual search for published work has also been done. The coronary heart disease studies were not restricted during the search by sample size because of the limited number of studies in selected countries. The study reviews the potential risk factors responsible for coronary heart disease globally. Smoking was highly prevalent among the United States and Pakistani populations, but hypertension and diabetes were more common in Tanzania and the United Kingdom. However, dyslipidaemia and obesity were common in almost all the selected countries. CVD risk factors are highly prevalent in some countries, varying socioeconomic, gender, and educational levels. Furthermore, there has always been a need for awareness in the public and educational programs for a healthy lifestyle, intake of nutritional food, and increased physical activity to improve health conditions and reduce the risk of cardiovascular diseases.

## Introduction and background

Atherosclerosis is the process of formation of plaque composed of cholesterol, fat, calcium, and other substances in the wall of large and medium-sized arteries causing diminished blood flow to an area of the body [1,2]. Moreover, atherosclerotic vascular disease can be classified into two arenas: a cardiovascular disease affecting the heart and peripheral blood vessels and cerebrovascular disease, which causes ischemic stroke in the brain [2-4]. Regrettably, cardiovascular and cerebrovascular diseases are the first and third leading causes of all deaths globally. Approximately 28% and a total of 247.9 deaths per one hundred thousand populations per year occurred from complications of ischemic heart diseases [5,6].

Coronary artery disease, also known as ischemic heart disease, occurs due to the formation of plaque, which is caused by the accumulation of cholesterol particles, and if the process continues, it may eventually reduce and block blood flow and decrease oxygen supply to the heart muscle [3-5]. Moreover, patients may not complain of chest pain or may have breathlessness and severe chest pain [7]. An estimated seven million people had ischemic strokes, which comprise 90% of all strokes, of which approximately 10% are caused by carotid artery stenosis [8]. Many studies attempted to evaluate the rate of asymptomatic carotid artery stenosis and unwanted neurologic events [9-12].

Atherosclerotic heart disease can be managed through medicines, surgical treatments, and lifestyle modifications, such as eating healthy, physical exercise, maintaining body weight, avoiding smoking and less salt intake [10-12]. The published study results stated that intake of more saturated fat could increase the risk of cardiovascular disease, a sedentary lifestyle increases weight, and hypertension is more likely to develop [8-14]. In recent studies, authors observed that physical activity could improve cardiorespiratory functions, and physical inactivity is a modifiable risk factor for developing CVD [5-10]. Researchers believe that women of young age who suffer from coronary heart disease have a higher risk of depression than men of the same age. The risk of developing heart disease in women was almost 50% in a whole life compared to men; the female had a high mortality rate after suffering from acute myocardial infarction (MI) [15-20]. 

This systematic review focuses on the prevalence of the potential risk factors of cardiovascular diseases, irrespective of age and gender, and its impacts on global public health. Furthermore, the study aims to draw attention to the need for health practitioners to ensure early interventions to prevent cardiovascular disease and its complications.

## Review

Methods and material

*Search Strategy* 

A literature search was conducted in the electronic database Medline, PubMed, Web of Science, Google Scholar, and the World Health Organization (WHO) website on all recent research work in the last five years (2015-2019) based on risk factors of CVD patients from a global perspective. Countries were selected from the European Union, Africa, Asia, and America to compare the global view of increasing risk factors of cardiovascular disease and gender differences. A strong association was found in those areas for cardiovascular disease burden among the non-communicable diseases (NCD) that significantly focus on global health strategy [14-18]. The keywords or MeSH (Medical Subject Headings) used during the search were ‘coronary artery disease, ‘coronary heart disease, ‘ischemic heart disease, ‘atherosclerosis’, ‘risk factors, ‘associated factors’, and ‘cardiovascular disease’.

Study Eligibility Criteria

The review included published papers covering both a healthy subject and a population with existing cardiovascular disease with different atherosclerosis risk factors during the literature search. All the abstracts were reviewed, and the selection of articles was made by the following eligibility criteria: This systematic review included cross-sectional and prospective cohort studies that evaluated major risk factors and family history associated with cardiovascular disease. The selected language was English for all the research articles, and each gender was given priority. Duplicate articles and papers with incomplete information, like conference proceedings, were excluded from the study.

Data Extraction

The initial search led to 506 publications from the existing published literature according to the eligibility criteria. After careful evaluation of the unique titles, abstracts, and availability of entire texts a total of 10 studies (Table 1) were selected that were relevant and fulfilled the study eligibility criteria. Data were extracted using structured data extractions sheets designed for this study and entered into a database.

**Table 1:** Brief description of included study with major findings

Author; Publication Year	Study type and duration	Study Population	Number of Individuals	Age and Gender	Major Findings
Sayeed et al. 2010 [19]	Cross-sectional study	Bangladeshi (Mymensingh)	6235	20-69 years (age ≥20Y)	Family history, older age (>45Y) has a significant risk of CVD
Nadeem M. et al. 2013 [21]	Observational study; April 2007 to December 2011	Pakistani	109	<45 Years (67 males & 42 females)	Most of the risk factors (smoking, hypertension, increased BMI, dyslipidemia, diabetes mellitus, increased abdominal girth, and family history) were equally prevalent in both gender except smoking which was less prevalent in females.
Gupta et al. 2012 [22]	Cross-sectional study	Indian	6198	Men 3426, Female 2772	Low socioeconomic, occupational status, and educational status had a higher risk of obesity, hyper-triglyceridemia, and CVD.
Roman et al. 2019 [23]	Cross-sectional study; April - July 2018	Tanzanian	100	66.8 years	Higher prevalence of hypertension, dyslipidemia, obesity, and physical inactivity.
Farrag et al. 2015 [24]	Cross-sectional study	Egypt	2895	19.5 ± 2.0 years	Most of the students were non-smokers. Young adults and adolescents were mostly obese and pre-hypertensive.
May et al. 2012 [25]	Cross-sectional, stratified, multistage probability sample survey (1999 - 2008)	United States	3383	12-19 years (Male 1771, Female 1612)	US adolescents carry a substantial burden of CVD risk factors, especially the youth who have an increased BMI.
Sani et al. 2010 [26]	Cross-sectional study; March - May 2006.	Nigerian	300	18-75 years (129 males & 171 females)	There was a high prevalence of CVD risk factors among the majority of the healthy adult Nigerian population.
Lee et al. 2011 [27]	Cohort study (1999-2008)	United Kingdom	32151	≥18 years	The incidence of CVD in the UK fell by 29%. The survival rate has increased due to the control of risk factors and improvement in drug treatments.
Weiss et al. 2018 [28]	Cross-sectional study (2012-2013)	Romanian	806	18-83 years (Males 36.8%)	The prevalence of CVD risk factors was high with abnormal lipid metabolism, smoking, and obesity. The Roma population has a high CVD risk burden.
Ramsay et al. 2014 [29]	A prospective study (1978–1980, 2010–2012)	British	1622	40-59 years, 71-92 years	CVD risk factors (HTN, high BP, obesity, diabetes) were associated with the older age group people.

**Figure 1:**
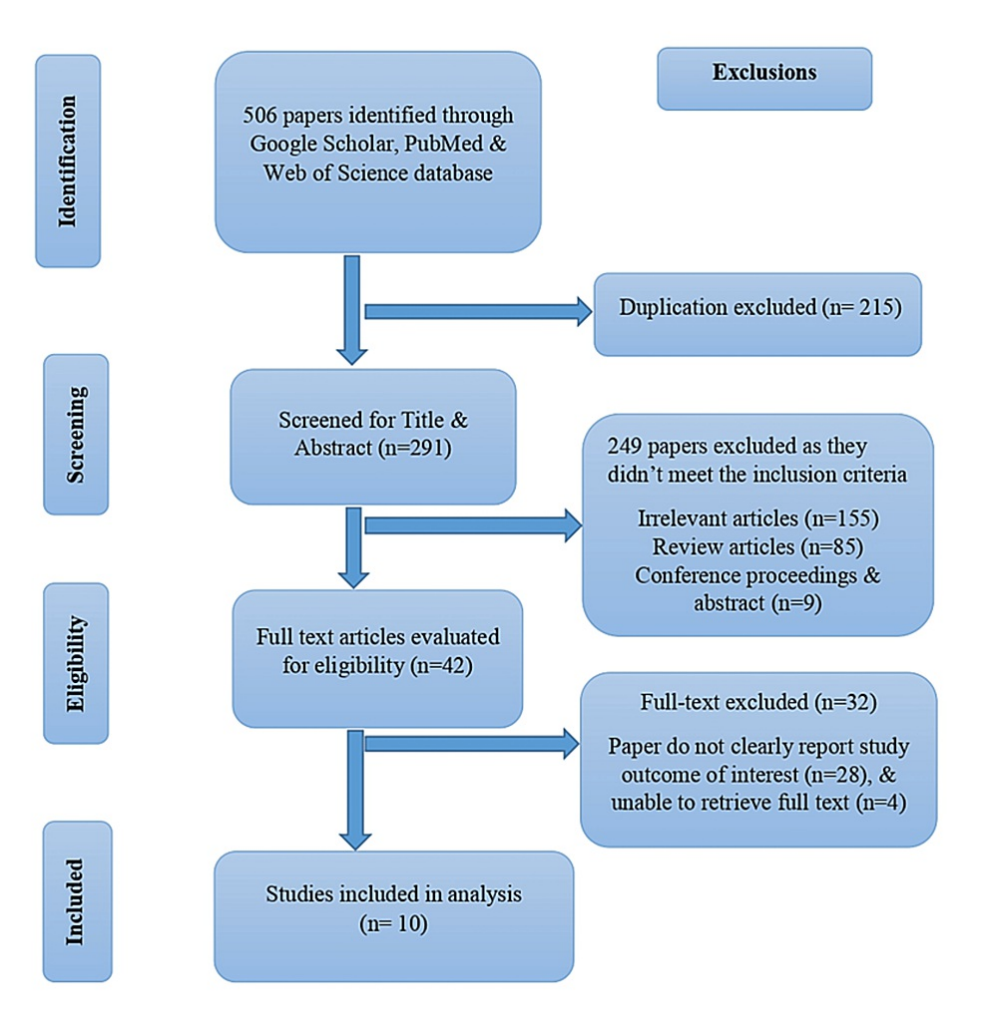
PRISMA flow diagram of the study PRISMA: Preferred Reporting Items for Systematic Reviews and Meta-Analyses

This systematic review was conducted according to the Preferred Reporting Items for Systematic Reviews and Meta-Analyses (PRISMA) statement and the Meta-Analysis of Observational Studies in Epidemiology (MOOSE) guidelines for our search strategy. The medical subject headings (MeSH) terms were utilised to find the preceding search terms in PubMed and Embase databases. The literature search covers additional references from the thesis or dissertation repositories, preprint servers, and manual searching of the reference lists from the preferred articles. Furthermore, the reasons for exclusion were duplication, irrelevant and review articles, articles with inadequate information, and reports we could not retrieve the full text. The cardiovascular disease studies were not restricted during the search by sample size because of the limited number of studies in selected countries. A PRISMA diagram explained the study sampling (Figure 1).

The extracted data from each database covered details of the published paper-like year of publication, type of the study, number of cohorts, sociodemographic variables, risk factors, and a global view of heart disease with the relation of different ethnic groups in various countries. Table 2 shows the prevalence of risk factors among included study population in percentages.

**Table 2:** Prevalence of risk factors among included study population (in percentages) Note: BMI- Body mass index; HTN- Hypertension; TG- Triglycerides; LDL- Low-density lipoproteins; HDL- High-density lipoproteins

Risk Factors vs Authors	Smoking (%)	Diabetes (%)	Hypertension (%)	Dyslipidemia (%)	Obesity (%)	Family history (%)
Sayeed et al. 2010 [19]	Insignificant	7.2%	7.1%	-	Increased BMI (19.4%) <23year (7.2%); ≥23 year (8.7%)	Significant association.
Nadeem et al. 2013 [21]	Overall 45.9% Males (43.1%); Females (2.8%)	18%	37%	33%	Increased BMI (63.3%)	43%
Gupta et al. 2012 [22]	6.9%	15.7%	31.6%	High level in Cholesterol (25%); & TG (36.9%); Low HDL (34.5%)	Increased BMI ≥25kg/m2 (42.9%); ≥30kg/m2 (11.6%)	-
Roman et al. 2019 [23]	Insignificant (only male)	Urban (5%); & Rural area (2%)	65%	High LDL (65%); Low HDL (79%)	Obese (39%); Overweight (36%); (Female > male)	53%
Farrag et al. 2015 [24]	>90% were non-smokers.	0.4%	2.1%	High cholesterol (8.3%)	Obese (10.7%); Overweight-27.9%	-
May et al. 2012 [25]	-	8%	2%	Increased LDL (6%); Decreased HDL (22%)	Obese (32%); Overweight (28%)	-
Sani et al. 2010 [26]	4.7%	5.3%	25.7%	High level in Cholesterol (28.3%); LDL (25.7%); & TG (15%); Low HDL (59.3%)	Increased BMI (>30 kg/m2) (21.3%); Male (10.9%); & Female-(29.2%)	-
Lee et al. 2011 [27]	44.2%	12%	65%	High cholesterol (38.7%)	Increased BMI (5.13%); Male (4.6%); & Female-(5.6%)	-
Weiss et al. 2018 [28]	63.02% including ex-smokers	15.1 % (new) & 75.4% (old) case	Uncontrolled HTN (33.6%)	High level in -Cholesterol (53.97%); LDL (66.87%); & TG (31.51%); Low HDL (22.7%)	Obesity (50.99%)	-
Ramsay et al. 2014 [29]	~65%	Frail (27%); Pre-frail (15%); & Not frail (14%)	Frail (78%); pre frail (74%); & not frail (65 %)	High LDL- Frail (6%); pre frail (8%); & not frail (11%). Low HDL- Frail (20%); pre frail (15%); & not frail (11%)	Increased BMI (≥30 kg/m2) Frail (24%); Pre frail (21%); Not frail (16%)	-

Results

A nationwide survey of the rural population in Bangladesh evaluated subjects aged 20 to 69 and found a higher prevalence of CVD and coexistence of multiple risk factors than the Japanese and Chinese populations [19,20]. In Pakistan, Nadeem and coworkers studied ischemic heart disease (IHD) patients and the observed age limit below 45 years, where the common risk factors were increased BMI, uncontrolled diabetes, hypertension, family history, increased cholesterol, obesity, and smoking history [21]. Moreover, an Indian study concluded a strong association between low socioeconomic status, less education and less occupational status, increased obesity, imbalanced lipid profile, smoking, and less physical activity [22].

According to the researchers, potential risk factors for coronary artery diseases are hypertension, obesity, and physical inactivity [23-25]. A study among the Nigerian population predicted an increase in CVD risk factors and mortality in developing countries linked with rising urbanization and socio-demographic changes with an age range from 18 to 75 years [26]. The UK highlights the importance of drug treatment in reducing the cardiovascular risk factors that helped decrease the incidence of stroke [27]. The risk factor for cardiovascular diseases is changing with the global trend, and this study will be elaborated on potential risk factors: smoking, diabetes, hypertension, dyslipidaemia, obesity, and family history.

Smoking: The prevalence of smoking has been addressed in nine of the studies, and the rates of smoking were 45.9% [21], 90% [24], 6.5% [22], and 4.7% [26]. Although smoking was an insignificant risk factor for CVD in Bangladesh and Tanzania, the USA, Pakistan, and Romania have significantly higher percentages of heavy smokers, about 44.2% [25], 43.1% [21], and 63.02% [28], respectively with the highest prevalence of ~80% among Nigerian male smokers [26].

Diabetes: This study addressed the rates of diabetes in the ten existing studies, and the overall prevalence was 5-27% among different nations. Recent studies observed a higher prevalence of diabetes mellitus in Pakistan (18%) [21], India (15.7%) [22], Romania (15.13%) [28], and the UK (12%) [27]. However, diabetes as a risk factor for CVD was almost controlled and found only in 5.3% and 0.4% of Nigerian and Egyptian people, respectively [24,26]. The prevalence of diabetes in the US increased from 9% to 23% over the last decades [25]. Although studies in India, Pakistan, and Romania showed that men had a higher prevalence of diabetes than women, in a study in Tanzania, females were more affected than males. [21-23,28].

Hypertension: The rate of hypertension was highest, approximately ~65% among males from Tanzania and British [23,27,29]. Several studies observed that increased age (more than 45 years) was significantly associated with increased blood pressure [23,29]. In comparison to Egypt and the United States [24,25], Bangladeshi people carry a slightly higher risk (2% vs 7%) of developing hypertension [19]. Across all the studies, the prevalence of hypertension among males and females were 20.2% vs 16.5%, 32.5% vs 30.4% and 25% vs 16.4% among Pakistani, Indian, and Nigerian population [21,22,26].

Dyslipidaemia: In India, total cholesterol was comparatively low, about 25%, but had raised LDL in 36.9% of cases, significantly higher than the Nigerian population [26]. Nonetheless, another Indian study found that 52% and 65% of males and females had low HDL, compared to 33% of the Pakistani population [21,22]. In Tanzania, high LDL and low HDL were in 65% and 79% of the population, respectively, and both abnormalities were more common in the female population [23]. Moreover, In Egypt, raised cholesterol was found in only 8.3% of cases, a minimum range, and no other abnormality has been detected [24]. About 39%, 22%, and 6% of patients in the UK and the United States had increased cholesterol, low HDL, and high LDL, respectively [25,27].

Obesity: In Bangladesh, the mean BMI was 19.4 kg/m^2^, and about 7.2% and 8.7% population aged <23 and ≥23 years, respectively, had obesity [19]. However, the prevalence of obesity was significantly higher in Pakistan (63.3%) and lowest in the U.S. (5.13%) [21,25]. Also, the prevalence of obesity in females was found to be lowest in the U.S. (5.6%) [25] and higher in Nigeria (29.3%) [26]. In India, approximately 43% and 12% of the population had BMI ≥25kg/m^2^ and ≥30kg/m^2^, respectively, and females are more prone to have higher BMI than men [22]. Nonetheless, in Egypt, about 11% and 28%, and in Tanzania, 39% and 36% were obese and overweight, respectively, and obesity was more common in females [23,24]. In Nigeria, a total of 21.3% of cases had BMI >30 kg/m^2^, and females were significantly higher (29.2%) than males (10.9%) [26]. In Romania, obesity was 51% population, and the male and female ratio was almost the same [28]. In Great Britain, about 20% of men in the age group of 71-92 years had BMI ≥30 kg/m^2^, and based on frailty scoring high BMI, British men were subgroups into frail (24%), pre-frail (21%), not frail (16%) [29].

Family history: Albeit positive family history is a known risk factor for CVD, recent studies from Tanzania, Pakistan, and Bangladesh observed that ~53%, ~43%, and ~4% population had a positive family history of coronary heart disease [19,21,23].

Discussion

Global Health Impact

The World Health Organization (WHO) has defined average weight as a body mass index (BMI) between 18.5 and 24.9 kg/m^2^, overweight as BMI between 25 and 29.9 kg/m^2^, and obesity as BMI ≥30 kg/m^2^ [30]. In the new global economy, there has been a declining trend in communicable diseases and malnutrition; but the cardiovascular disease has become a central issue for people of high, middle, or lower-class socioeconomic statuses [31,32]. Moreover, low socioeconomic people reported the most harmful CVD risk factors due to less access to advanced treatments, less education, and poor economic situation [30-34]. In the United Kingdom, over 11.6 years of follow-up of 1.2 million women, there was a significant relationship between increased risk of CVD and females at the age of menarche [35]. Over the two decades, Sub-Saharan Africa has been experiencing relatively decreased CVD burden levels; however, this area's mortality has increased steadily, precisely due to hypertensive heart disease [9,20]. A recent study shows that the women population had increased physical activity, but the incidence of hypertension (HTN), obesity, and diabetes are increasing daily [15-22,30-35].

Future Directions in Measuring the Global Cardiovascular Disease Burden

In a recent study, authors observed more influence of behavioural factors than preventive medicine in managing cardiovascular diseases [10-15]. According to current study results, smokers who did not follow a healthy diet or active life like regular exercise were more likely to be given medication like antiplatelet, statin, and anti-hypertensive, similar to other existing study results [25-30]. Furthermore, recent studies suggested that mortality related to cardiovascular disease can be low if there is a reduction in specific risk factors like cholesterol, HTN, and obesity [19-24,30-34]. Albeit physical exercise and a healthy diet lessen the risk of CVD, heavy smokers with a healthy diet and exercise still had a 3.8-fold increased risk of CVD, in concordance with existing articles [35-38]. 

Recent works show that health policymakers should not be waiting for a perfect epidemiological study that may guide them to certain decisions anymore [28-32]. The US health organizations guidelines enforced the following issues to decrease the burden of cardiovascular disease, prevention of disease and promotion of health concerning smoking, improve public health policy and health advocacy on adverse effects of increased salt in food, unhealthy eating habits, physical inactivity; and also reduction of the CVD risk factors especially hypertension [34]. Existing research recognizes that the critical role played for the forthcoming measurements of global burden in CVD will be increased, informed by new vital registration systems [35-40]. Sustainable development goals, along with human resources, technical capacity, infrastructures, and funding, will be in need, particularly in LMICs [38-45].

Strength and limitations

This study is based on a global population covering different ethnic groups of the population, which compares all age groups and gender. Although there has been some lack of presenting data at some national levels, there was an identical pattern of risk factors for CVD in most countries, especially the region that shared the same culture, environment, cuisine, and lifestyle. Moreover, we only included English-language papers, which may preclude important study findings published in other languages. Albeit, this systematic review was based on globally acceptable major cardiovascular risk factors, the availability of the papers was limited to some parts of Asia, Europe, Africa, and the United States. Moreover, we could not perform the sensitivity analyses due to insufficient data, and primary authors have not been contacted, which may give some unadjusted results to the review.

## Conclusions

The potential risk factors for CVDs are highly prevalent in different geographical regions, varying with the nation's socioeconomic, gender and educational levels. Furthermore, atherosclerosis and hypertensive heart disease are significant global health problems, and this review recommends a combined and intensive multi-disciplinary team approach at the population and individual level to diminish the burden of CVDs in all regions. Thus, this review could help to improvise practice and policy and provide the newest insight from a large group of data syntheses to reduce the global burden of cardiovascular disease morbidity and mortality.
